# Editorial: Critical reviews and perspectives in *Nucleic Acids Research*

**DOI:** 10.1093/nar/gkac598

**Published:** 2022-07-08

**Authors:** David R Corey, Helle D Ulrich

Articles that summarize knowledge are important contributions to science. Such articles should provide a single authoritative source of information relative to a particular scientific field or question. At *Nucleic Acids Research*, the editorial team realizes that rapid growth of potentially relevant papers increases the challenge of preparing review articles that will appeal to our broad readership.

Recognizing these challenges, we have recently revised our guidelines (https://academic.oup.com/nar/pages/criteria_scope) to make our expectations clear. First and foremost, we expect that authors demonstrate a passion for explaining their topic. Authors should see an important unmet need, a topic where researchers lack up-to-date syntheses of the best information on a subject. Authors should also have deep expertise. The scientific literature is vast. Not all papers are equal in quality. Authors should be able to identify the best, most rigorous studies. They should provide guidance to readers on the hallmarks of rigorous (or less than rigorous) research. Manuscripts should provide some guidance about challenges for future research.

We welcome expert opinion, and we hope that authors will enjoy sharing their hard-earned authority to judge with the community. We note, however, that we continue to welcome more traditional review articles that summarize key findings in a field. Implicit in such papers, however, is the fact that authors possess the expertise to identify the most reliable data and set it into context. We are looking for papers that weave a field together, not present it as a series of unconnected findings. Critical Review and Perspective articles can be commissioned by the editorial team, but we also welcome suggestions from authors. We are happy to provide feedback to authors if we feel that a potential submission is not ready but might have the potential to be improved.

In this issue, we present the first commissioned Critical Review and Perspective. This Perspective is not a typical *NAR* review, because rather than focus on a particular scientific question, it focuses on the scientific process itself. In 1989, Dr Stanley Crooke left his position as head of Research and Development at Smith Kline Beckman to start Ionis Pharmaceuticals, a company dedicated to a daring new concept—turning synthetic oligonucleotides into drugs. There were many scientific challenges. How can a large negatively charged synthetic molecule enter cells? How can it be made economically on large scale? How can it be chemically modified for biological activity? In 1989, the basic science underlying the field was primitive—the first automated small scale synthesizers had begun to enter laboratories just a few years previously. Nothing was known about the pharmacological properties of oligonucleotides. With so little scientific background, identification of appropriate disease targets was speculative.

Dr Crooke dedicated over thirty years to tackling these problems at Ionis. The teams he has helped supervise have created several approved drugs. One drug, Spinraza, has had a major impact on the treatment of spinal muscular atrophy, providing hope to patients and their families and demonstrating that nucleic acids drugs can have a major impact. While pursuing drug development, Dr Crooke has led his own research group and encouraged Ionis employees in general to publish their work and support basic science through collaborations.

The question of how to address difficult scientific problems is one that is often faced by the authors submitting work to *Nucleic Acids Research*. The question of rigor is one that *NAR* editors and reviewers face when evaluating thousands of submissions each year. We hope that Dr Crooke's perspective will be a useful guide for those seeking to understand how to balance rigorous basic science with the practical demands of drug development. We expect that this Perspective will not only inspire scientists in industry, but also in academia.

